# The usefulness of point-of-care ultrasound in dehydrated patients in a pediatric emergency department

**DOI:** 10.1186/s13089-023-00354-1

**Published:** 2024-02-21

**Authors:** Tommaso Bellini, Benedetta Chianucci, Matteo D’Alessandro, Margherita Ricci, Maria Grazia Calevo, Silvia Misley, Emanuela Piccotti, Andrea Moscatelli

**Affiliations:** 1 Pediatric Emergency Room and Emergency Medicine Unit, Emergency Department, IRCCS Istituto Giannina Gaslini, Via G. Gaslini 5, 16147 Genoa, Italy; 2 Pediatric and Neonatology Unit, San Paolo Hospital (Savona), IRCCS Istituto Giannina Gaslini, Genoa, Italy; 3 Epidemiology and Biostatistics Unit, Scientific Directorate IRCCS Istituto Giannina Gaslini, Genoa, Italy; 4 Department of Neuroscience, Rehabilitation, Ophthalmology, Genetics, Maternal and Child Health (DINOGMI), University of Genoa, Genoa, Italy; 5 Neonatal and Pediatric Intensive Care Unit, Emergency Department, IRCCS Istituto Giannina Gaslini, Genoa, Italy

**Keywords:** Point-of-care ultrasound, Pediatric emergency department, Dehydration, Acute gastroenteritis

## Abstract

**Backgrounds:**

Dehydration is among the most common causes of Pediatric Emergency Department admission; however, no clinical signs, symptoms, or biomarkers have demonstrated sufficient sensitivity, specificity, or reliability to predict dehydration.

**Methods:**

We conducted a prospective, monocentric, observational study at Giannina Gaslini Hospital, a tertiary care pediatric hospital. Our study aimed to compare inferior vena cava ultrasound measurement with volume depletion biomarkers to understand if point-of-care ultrasound could help grade, evaluate, and better manage dehydration in children presenting to the pediatric emergency department. We enrolled patients under the age of 14 who required blood tests in the suspect of dehydration; for each patient, we collected values of venous pH, natremia, bicarbonatemia, uric acid, chloremia, and blood urea nitrogen. For each patient, we performed two ultrasound scans to calculate the Inferior Vena Cava/Aorta area ratio and to assess the IVC collapsibility index; moreover, we described the presence of the “kiss sign” (100% IVC walls collapsing during the inspiratory phase).

**Results:**

Patients with the “kiss sign” (25/65 patients, 38.5% of the total) showed worse blood tests, in particular, uric acid levels (*p* = 0.0003), bicarbonatemia (*p* = 0.001) and natriemia (*p* = 0.0003). Moreover, patients with the “kiss sign” showed a high frequency of ≥ 2 pathological blood tests (*p* = 0.0002). We found no statistical significant difference when comparing the IVC/Ao ratio and IVC-CI with the considered blood tests.

**Conclusions:**

The “kiss sign” seems to be related to worse hydration state, whereas IVC/Ao and IVC-CI are not. In an emergency setting, where physicians must take diagnostic-therapeutic decisions quickly, the presence of the “kiss sign” in patients suspected to be dehydrated can be a helpful tool in their management.

## Introduction

Dehydration is among the most common causes of admission to the Pediatric Emergency Department (PED) [1]. Therefore, an accurate assessment of dehydration status is crucial to ensure targeted treatment and prevent morbidity and mortality in children with gastroenteritis. Several attempts were made to establish the best clinical or biochemical marker of dehydration, such as the Clinical Dehydration Scale (CDS) proposed by Friedman [2], serum levels of sodium, potassium, chloride, bicarbonate, urea, pH and albumin. However, no clinical sign, symptom, or biomarker has demonstrated sufficient sensitivity, specificity, or reliability in predicting dehydration; this is mostly related to multiple interfering factors with previous biomarker measurement but is also depending on the leading cause of dehydration (diarrhea, vomiting, polyurea, reduced thirst, ecc.) [3–8].

The most accepted standard criteria to determine the grade of volume depletion is the percentage of weight loss; however, the pre-illness weight is rarely available in the acute care setting [6]. In this context, there is a pressing need to develop a fast, non-invasive, and objective tool to accurately assess the volume status of dehydrated children [1].

Bedside ultrasonography may be helpful for this purpose. In the pediatric population, an increasing number of studies have introduced ultrasound measurement of the inferior vena cava (IVC) as a non-invasive diagnostic tool for intravascular volume evaluation and as a surrogate for central venous pressure [9, 10]. Moreover, point-of-care ultrasound (POCUS) has been proposed as innovative method to establish fluid responsiveness in critical care setting through respiratory variation in inferior vena cava diameter [11–13]. Use of bedside ultrasonography is undoubtedly promising mainly because is a low-cost, reproducible and easy to perform technique: unfortunately, the current literature, considering the potential role of IVC, shows extreme heterogeneity, making it challenging to validate IVC ultrasonography in predicting fluid depletion [14].

Our study aimed to compare IVC measurements with volume depletion biomarkers to understand if POCUS could help grade, evaluate, and better manage dehydration in children presenting to the PED.

## Materials and methods

This prospective, monocentric, observational pilot study was conducted in the PED of Giannina Gaslini Children’s Hospital, a tertiary care pediatric hospital, with approximately 35,000 PED visits/year, from 1st July to 31st October 2022.

We enrolled patients aged ≤ 14 who accessed the PED that required blood tests in the suspect of moderate-to-severe dehydration based on clinical-anamnestic evaluation (oral rehydration failure, high number of vomiting/diarrhea episodes, reduced skin turgor, poor capillary refill, oliguria, and/or lethargy) [15, 16]. For each patient, we collected data on venous pH, uric acid, natremia (Na), bicarbonatemia (HCO_3_), and blood urea nitrogen (BUN). Exclusion criteria included known cardiac, liver, or kidney diseases; preterm birth; life-threatening conditions. Notably, all patients were treated according to our existing internal protocol for dehydrated children [17]. In addition, POCUS was performed before any oral or intravenous fluid administration.

In the supine position, the patients were scanned using SonoAce-R3 (*Samsung* Medison, *South Korea*) or My Lab 30Gold (*Esaote, Italy*) machines equipped with convex probes. The operators consisted of two pediatric emergency physicians with US certificates. We performed two ultrasound scans per patient [1, 9, 12].

**Fig. 1:**
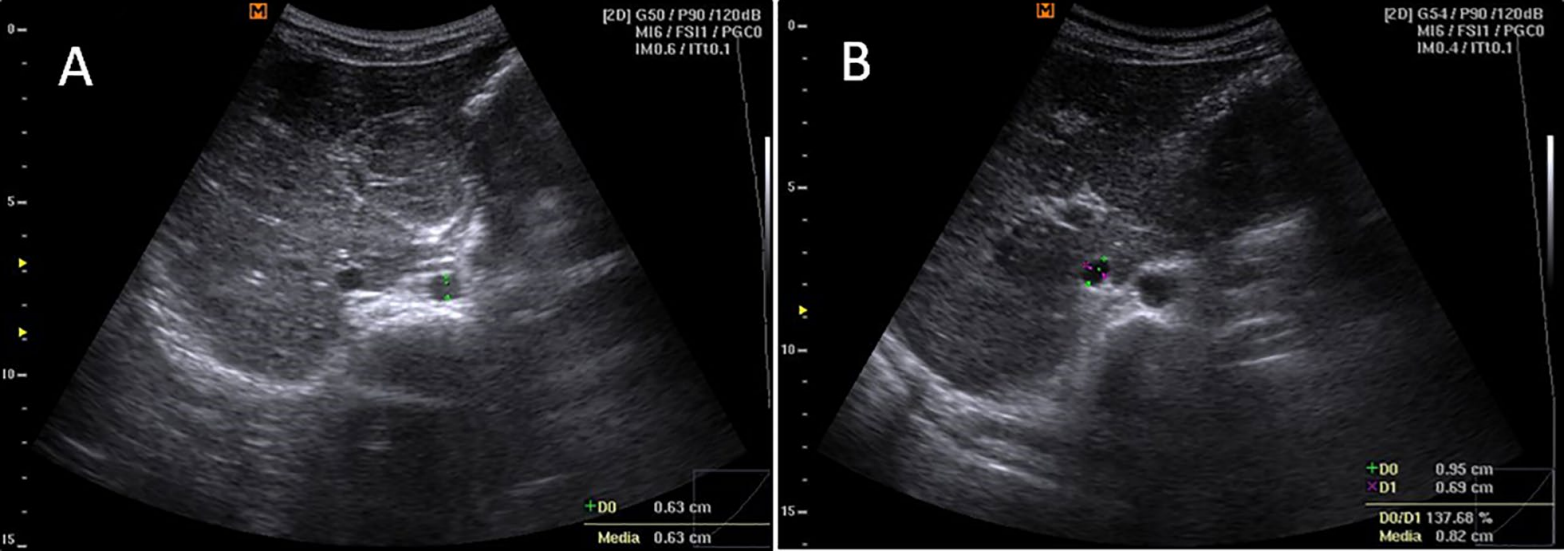
Transverse plane scan, short-axis view, B-mode. **A** shows the anteroposterior measurement of the aorta during systole, and **B** shows the anteroposterior and latero-lateral measurements of the inferior vena cava during exhalation

The first scan was performed in the transverse plane by placing the probe over the abdomen just below the xiphoid bone. Here, we visualized the aorta (Ao) and IVC in the cross-section, measuring their maximal calipers during systole for Ao and exhalation for IVC in US B-mode (Fig. 1). The IVC cross-sectional area was calculated as $3.14*\frac{D}{2}*\frac{d}{2}$ where D is long axis and d is short axis of the IVC. The Ao cross-sectional area was calculated as $3.14*{r}^{2}$ where r is the radius of the aorta. Finally, we calculated the IVC/Ao area ratio.

**Fig. 2:**
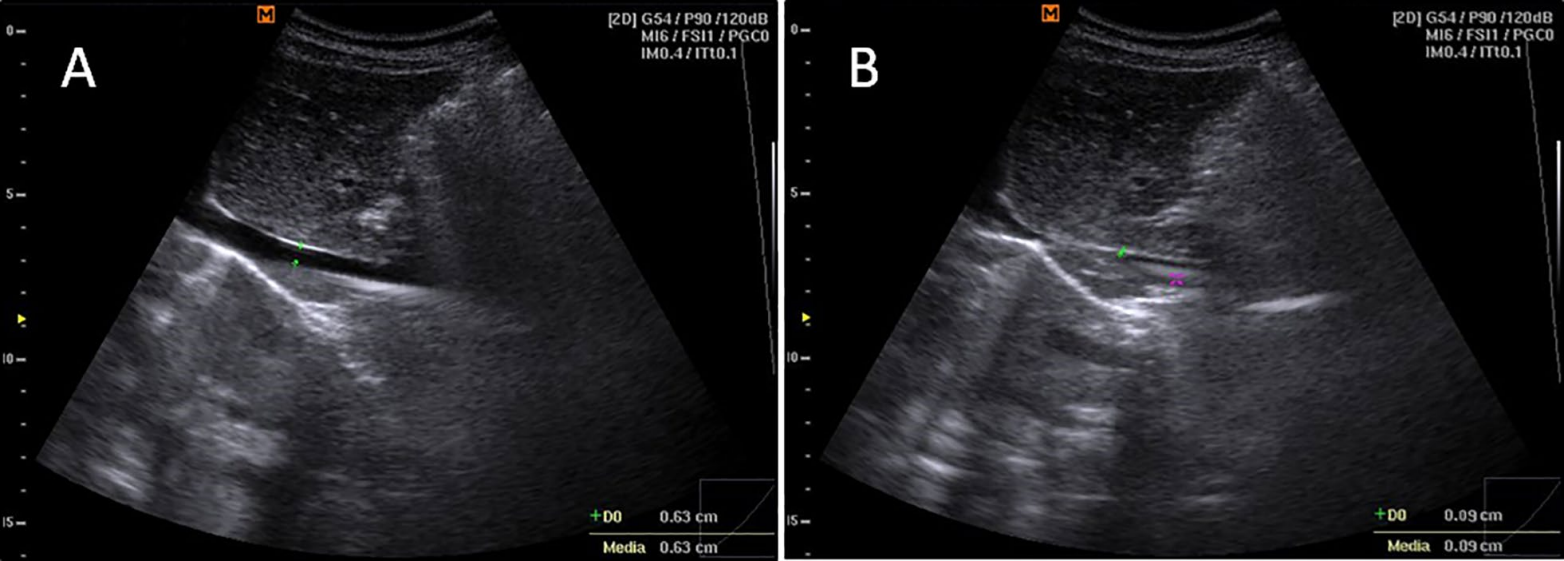
Longitudinal plane scan, long axis view, B-mode. **A** shows the inferior vena cava antero-posterior diameter during exhalation and **B** shows the presence of the “kiss sign” (~ 100% IVC walls collapsing during the inspiratory phase)

The second scan was performed in the longitudinal plane, to assess IVC collapsibility during the respiratory phases and the presence of the “kiss sign” (100% IVC walls collapsing during the inspiratory phase) in the long-axis view (Fig. 2), recorded 2 cm before the atrial inlet.

**Fig. 3:**
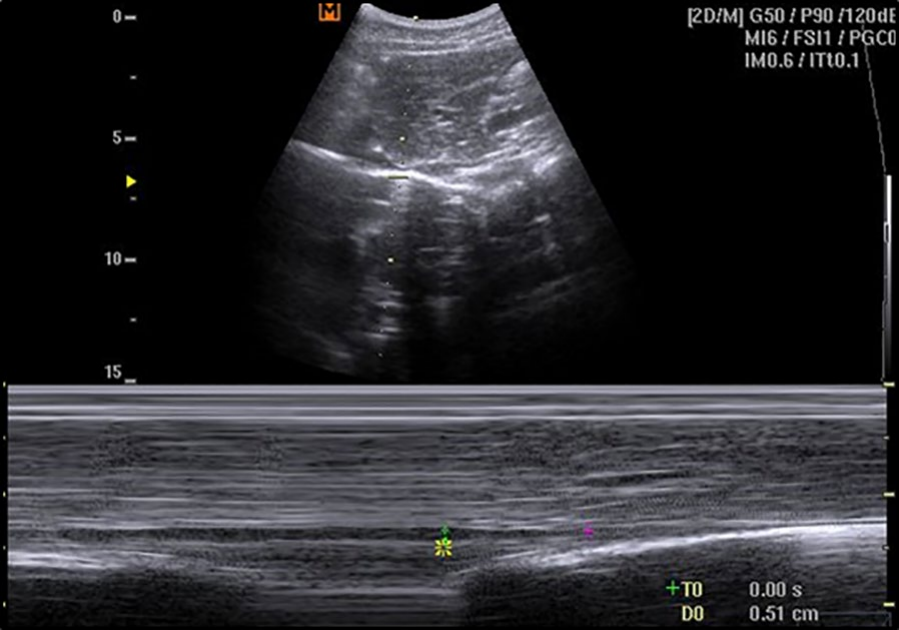
Longitudinal plane scan, long-axis view. M-mode “kiss Sign” recording

The diameters of the IVC were measured in B-mode and used to calculate the IVC collapsibility index (IVC-CI) using the formula: $100*\frac{IVC{\text{max}}- IVC min}{IVC max}$. Finally, we registered the image in the M-mode if the “kiss sign” was detected (Fig. 3). All the measures were conducted during spontaneous respiration, avoiding crying where feasible.

The patients were categorized into two groups based on the absence or presence of the IVC “kiss sign”; we further divided the patients into two other groups based on the number of pathological blood tests (< 2 or ≥ 2).

The study was approved by the Regional Ethics Committee (#0022726/22, June 2022).

### Statistical analysis

A descriptive analysis was performed, and the data were expressed as median and interquartile range (IQR) and absolute and relative frequencies for categorical variables. Comparisons between groups were made using non-parametric tests (Mann–Whitney U-test) for continuous variables. The association between categorical variables was assessed using χ^2^ or Fisher’s exact test. All *p*-values were calculated using two-tailed tests, considering a p-value less than 0.05 to be statistically significant. Statistical analysis was conducted using SPSS for Windows version 18 (SPSS Inc., Chicago, Illinois, USA).

## Results

Sixty-five patients were enrolled, thirty-three male (50.5%). Median age was 5.34 years (range 1.16–14.4). No age or sex differences were noted between the groups.

**Fig. 4:**
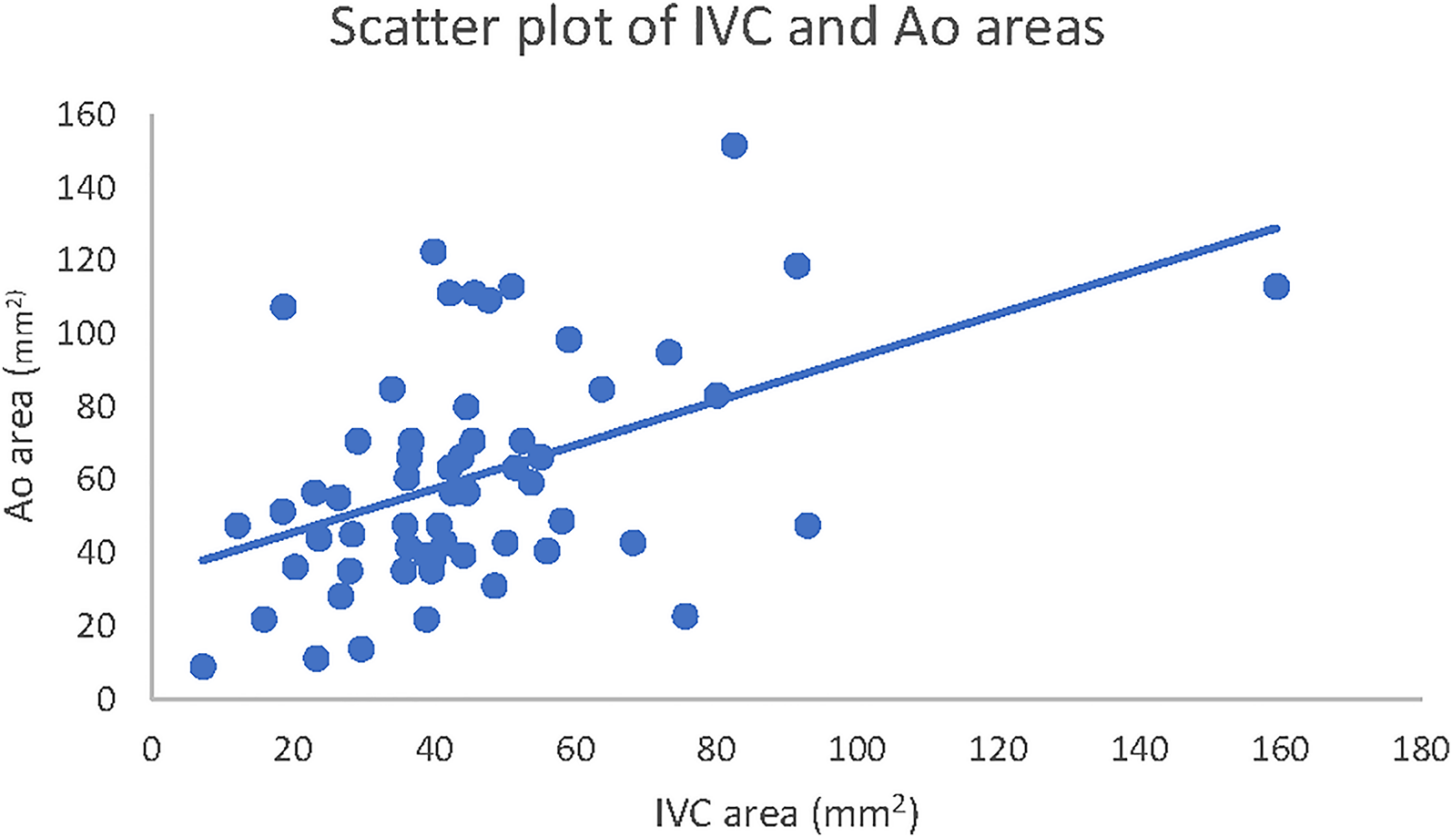
Scatter plot of IVC and Ao areas

**Fig. 5:**
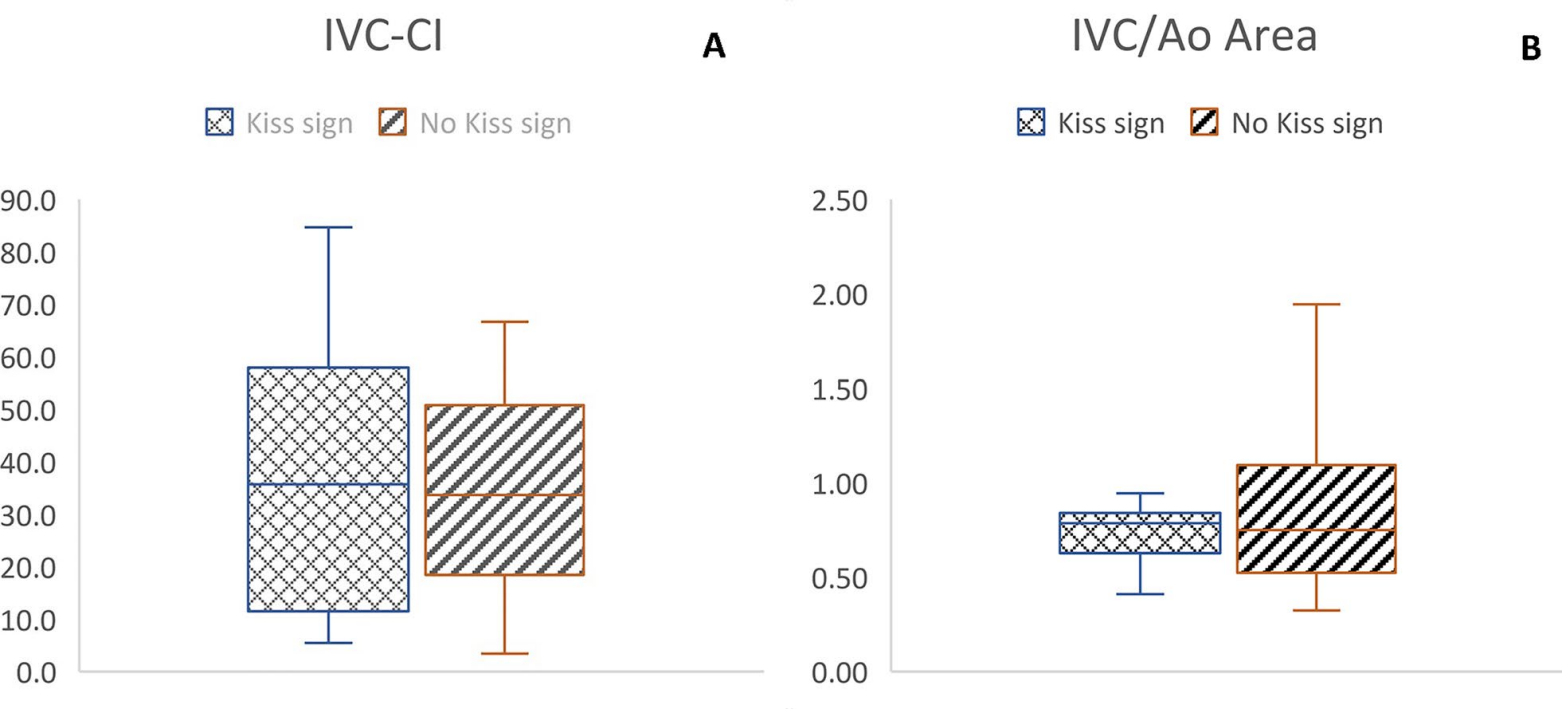
Box plot of IVC-CI (**A**) and IVC/Ao area (**B**) in “Kiss sign” patients and no “Kiss sign” patients

The “kiss sign” group (25/65 patients, 38.5% of the total) showed worse blood tests, in particular, uric acid (*p* = 0.0003), HCO_3_ (*p* = 0.001) and Na (*p* = 0.003), all in terms of absolute and pathological values. We also observed worse venous pH and BUN, albeit without statistical significance. Previous data are summarized in Table 1. We did not find any statistical significance when comparing the IVC/Ao ratio and IVC-CI with the considered blood tests (Table 2, Figs. 4, 5).

**Table 1:** Correlation between the presence/absence of the Kiss Sign and blood biomarkers

		Kiss sign No	Kiss sign Yes	*p* value
	*n* = 65 (100%)	*n* = 40 (61.5%)	*n* = 25 (38.5%)	
Sex*, m*	33 (50.5%)	21 (52.5%)	12 (48.0%)	0.72
Age, *years*	5.34 (3.87)	4.01 (3.49)	5.76 (4.11)	0.26
pH	7.33 (0.05)	7.35 (0.05)	7.33 (0.08)	0.9
pH < 7.31*, yes*	12 (18.5%)	7 (58.3%)	5 (41.7%)	1
Uric acid*, mg/dl*	6 (3.9)	4.35 (3.68)	8 (3)	**< *0.001***
Uric acid > 5 mg/dl, yes	39 (60%)	17 (43.6%)	22 (56.4%)	**< *0.001***
Na*, mEq/l*	135 (4)	135 (2.25)	132 (1)	**< *0.001***
Na < 135 mEq/l, yes	30 (46.1%)	11 (36.7%)	19 (63.3%)	**< *0.001***
BUN, mg/dl	35 (12)	35 (16.25)	37 (2)	< 0.08
BUN > 40 mg/dl*, yes*	9 (13.8%)	5 (55.6%)	4 (44.4%)	< 0.72
HCO_3_, mEq/l	17 (4.6)	19 (3.15)	15 (2)	***0.048***
HCO_3_ < 18 mEq/l, yes	35 (53.8%)	15 (42.9%)	20 (57.1%)	**< *0.001***

Bold and italic combined have been used for significant results (below 0.05)

Na, natremia; HCO_3_, bicarbonatemia; BUN, blood urea nitrogen. Sex and number of subjects with pathological biomarkers are expressed as absolute numbers and percentages; age and absolute biomarker values are reported as median and interquartile range

**Table 2:** Correlation between pathological biomarkers and POCUS measurements

	*n* (%)	IVC/Ao Median (IQR)	*p* value	IVC-CI Median (IQR)	*p* value
pH < 7.31, yes	12 (18.5%)	0.75 (0.38)	0.72	34.2 (47)	0.16
Uric Acid > 5 mg/dl, yes	39 (60%)	0.79 (0.31)	0.94	35.2 (43.6)	1
Na < 135 mEq/l, yes	30 (46.1%)	0.79 (0.3)	0.76	30.5 (44.4)	0.91
BUN > 40 mg/dl, yes	9 (13.8%)	0.75 (0.55)	0.47	40.8 (30.9)	0.53
HCO_3_ < 18 mEq/l, yes	35 (53.8%)	0.79 (0.26)	0.36	28.3 (42.6)	0.33
Total	65 (100%)	0.79 (0.42)		35.2 (42.2)	

IVC/Ao area ratio and IVC-CI are reported as median and IQR. IVC: Inferior Vena Cava; Ao: Aorta; CI: collapsibility index; IQR: interquartile range

Considering the number of pathological blood tests, we found no correlation between ≥ 2 pathological blood test group and a greater IVC-CI (*p* = 0.98) or IVC/Ao ratio (*p* = 0.72). However, we found a higher frequency of “kiss signs” in this group (*p* = 0.0002). These data are summarized in Table 3.

**Table 3:** Correlation between the number of pathological exams, kiss sign and POCUS measurements

		< 2 pathological exams	≥ 2 pathological exams	*p* value
	*n* = 65	*n* = 27	*n* = 38	
Kiss sign, yes	25 (38.5%)	3 (12%)	22 (88%)	**< *0.001***
IVC/Ao	0.79 (0.42)	0.77 (0.58)	0.79 (0.3)	0.72
IVC-CI	35.2 (42.2)	35.2 (37.7)	34.2 (43.7)	0.98

Bold and italic combined have been used for significant results (below 0.05)

IVC/Ao and IVC-CI are reported as median and IQR. IVC: Inferior Vena Cava; Ao: Aorta; CI: collapsibility index; IQR: interquartile range

## Discussion

To the best of our knowledge, our study is the first Italian ultrasound study comparing IVC measurement with volume depletion blood markers to understand whether POCUS could help evaluate and grade dehydration in children presenting to the PED.

Previous studies have shown that US evaluation of IVC could be a helpful tool to assess volume status in dehydrated children, owing to the need for more reliable clinical evaluation, particularly for younger patients.

The IVC/Ao ratio and IVC-CI are the most frequently used parameters; we investigated these indexes before fluid administration in children with signs and symptoms suggestive of dehydration but we found no statistical significance [18–20].

We introduced the M-mode detection of the “kiss sign” and our findings suggested that its presence may be predictive for worse blood tests, in particular a higher level of uric acid and a lower level of Na and HCO_3_, all related to worse hydration status, given the normal renal function of our subjects.

With the caution given by the small sample size, this result may suggest that the presence of the “kiss sign” in children who are probably dehydrated could advise clinicians to perform blood tests and consider intravenous rehydration; this finding seems to agree with the observation that healthy euvolemic children do not have 100% collapsibility of the IVC, which is instead commonly observed in patients with various degrees of dehydration [21, 22].

To our knowledge, only one study has compared IVC ultrasonography with blood tests [1]. In this case series, 124 children underwent measurement of IVC/Ao ratio and IVC-CI; they were correlated with worse blood markers, in particular HCO_3_ and C-reactive protein levels. However, there was no significant correlation between IVC/Ao ratio and IVC-CI and total white blood cell count, hemoglobin, hematocrit, glycemia, BUN, creatinine, uric acid, Na, K, venous pH, and lactate. These data can be partially comparable to ours, while other Authors suggest a correlation between IVC-CI and IVC/Ao ratios and the severity of dehydration [1, 6, 9, 11, 23]*.*

The current study has some limitations.

First, we included a convenience sample of patients when physicians were on the shift in our PED; thus, it could be challenging to understand whether our number approximates the general population of children with suspected dehydration. However, albeit low, this sample was similar to that reported previously [23]. Second, performing an IVC scan, even after rehydration, to assess the variation of the US indices and their improvement could have added value to the study; this has been done by Özkan et al., who showed that the IVC/Ao ratio and IVC diameters were significantly lower in moderate-to-severe dehydrated patients than in mildly dehydrated patients, both before and after fluid therapy, but improved after IV therapy [1]. Moreover, the observational design and the single center setting of the study are relevant limitations. Finally, the evaluation of the IVC can be performed only in cooperative patients, as crying children determine an abdominal pressure that invalidates the measurements of the IVC. Indeed, the relationship between respiratory effort and the caliber of the IVC has been extensively investigated only in certain clinical scenoarios, such as in a calmly spontaneously breathing patient or under fully passive mechanical ventilation. It is not known, however, how the IVC-to-respiratory cycle ratio varies when the effort for it is more than the baseline (for example, during crying, sobbing breath, or in a partially assisted ventilated patient).

## Conclusions

In conclusion, this study could be encouraging, as POCUS could soon become a turning point in managing patients with suspected dehydration, helping clinicians classify and treat this condition.

The observation of the “kiss sign” may be performed at a glance, thus resulting in a more immediate and, therefore, more suitable emergency ultrasound evaluation of the patient, in contrast to the IVC/Ao ratio and IVC-CI, which instead require calculation.

In an emergency setting, where the PED physician has to take diagnostic-therapeutic decisions in a minimal time, the presence of the “kiss sign” in dehydrated patients can be an encouraging aid in their management. However, further and more significant numbers are needed to confirm these results.

## Data Availability

The datasets generated and/or analyzed during the current study are not publicly available due to privacy policy but are available from the corresponding author on reasonable request.
